# Peer-to-peer tele-consultative services for critical care, Afghanistan, Kenya, Pakistan, United Republic of Tanzania

**DOI:** 10.2471/BLT.23.290926

**Published:** 2024-12-04

**Authors:** Asad Latif, Huba Atiq, Mareeha Zaki, Syeda A Hussain, Ammarah Ghayas, Omer Shafiq, Ali A Daudpota, Qalab Abbas, Shabina Ariff, Muhammad A Asghar, Muhammad F Khan, Muhammad H Khan, Naveed Rashid, Amber Sabeen, Muhammad Sohaib, Hameed Ullah, Tahir Munir, Mohammad M Hassan, Kiran Sami, Syed K Amin, Zainab Samad, Adil Haider

**Affiliations:** a Department of Anaesthesiology, Aga Khan University, Stadium Road, P.O. Box 3500, Karachi 74800, Pakistan.; b Dean’s Office, Medical College, Aga Khan University, Karachi, Pakistan.; c Department of Paediatrics & Child Health, Aga Khan University, Karachi, Pakistan.; d Department of Medicine, Aga Khan University, Karachi, Pakistan.

## Abstract

**Objective:**

To develop a tele-intensive care service providing peer-to-peer teleconsultation for physicians in remote and resource-constrained health-care settings for treatment of critically ill patients, and to evaluate the outcomes of the service.

**Methods:**

The Aga Khan University started the coronavirus disease 2019 (COVID-19) tele-intensive care unit in 2020. A central command centre used two-way audiovisual technology to connect experienced intensive care specialists to clinical teams in remote hospital settings. The service, always available, used messaging applications and telephone calls. Coverage was later extended to other medical, neonatal, paediatric and surgical patients requiring critical care.

**Findings:**

Between June 2020 and December 2023, the service provided 6014 teleconsultations to manage 1907 patients in 109 medical facilities, mostly in Pakistan and also Afghanistan, Kenya and United Republic of Tanzania. Of the 1907 patients, 652 (34.4%) had COVID-19 and 1244 (65.6%) had other illnesses. The mean duration of teleconsultations was 14.5 min. Of 581 patients for whom outcome data were available, 204 (35.1%) died. Multivariate multinomial logistic regression showed the odds of death decreased with increased number of consultations (> 3) per patient (adjusted odds ratio (aOR): 0.28; 95% confidence interval, CI: 0.16–0.48), and increased number of recommendations (≥ 5) per consultation (aOR: 3.09; 95% CI: 1.08–8.84).

**Conclusion:**

Our tele-intensive care service helped manage critically ill patients in regions where intensive care had not previously been available. While research on the clinical impact of this model is needed, decision-makers should consider its use to increase provision of critical care in remote and resource-constrained health-care settings.

## Résumé

**Objectif:**

Mettre en place un service à distance pour soins intensifs fournissant une téléconsultation de pair à pair pour les médecins d’établissements de santé éloignés et présentant des ressources limitées dans le cadre du traitement de patients gravement malades et évaluer les résultats de ce service.

**Méthodes:**

En 2020, l’Université Aga Khan a mis en place le service de téléconsultation en soins intensifs dans le cadre de la maladie à coronavirus 2019 (COVID-19). Un centre de commandement central a mis à profit une technologie audiovisuelle bidirectionnelle pour mettre en relation des spécialistes expérimentés en soins intensifs et des équipes cliniques situées dans des hôpitaux éloignés. Ce service, toujours disponible, a recours à des applications de messagerie et à des appels téléphoniques. La couverture a ensuite été étendue à d’autres patients dans les secteurs néonatal, pédiatrique, chirurgical et de médecine générale nécessitant des soins intensifs.

**Résultats:**

Entre juin 2020 et décembre 2023, le service a fourni 6014 téléconsultations pour prendre en charge 1907 patients dans 109 établissements médicaux, principalement au Pakistan, mais aussi en Afghanistan, au Kenya et en République-Unie de Tanzanie. Sur ces 1907 patients, 652 (34,4%) étaient atteints de COVID-19 et 1244 (65,6%) d’autres maladies. La durée moyenne des téléconsultations était de 14,5 minutes. Sur les 581 patients pour lesquels des données sur les résultats étaient disponibles, 204 (35,1%) sont décédés. Une régression logistique multinomiale multivariée a mis en évidence la diminution du risque de décès parallèlement à l’augmentation du nombre de consultations (> 3) par patient (odds ratio ajusté (aOR): 0,28; intervalle de confiance (IC) à 95%: 0,16–0,48), ainsi que l’augmentation du nombre de recommandations (≥ 5) par consultation (aOR: 3,09; IC à 95%: 1,08–8,84).

**Conclusion:**

Notre service de téléconsultation pour soins intensifs a contribué à la prise en charge de patients gravement malades dans des régions où les soins intensifs n’étaient pas disponibles auparavant. Bien qu’une analyse de l’impact clinique de ce modèle s’impose, les décideurs devraient envisager de l’appliquer afin d’augmenter la fourniture de services de soins intensifs dans les régions éloignées et aux ressources limitées.

## Resumen

**Objetivo:**

Desarrollar un servicio de teleasistencia en cuidados intensivos que ofrezca teleconsultas entre iguales a médicos de entornos sanitarios remotos y con recursos limitados para el tratamiento de pacientes en estado crítico, y evaluar los resultados del servicio.

**Métodos:**

En 2020, la Universidad Aga Khan inició la unidad de teleasistencia en cuidados intensivos para la enfermedad por coronavirus 2019 (COVID-19). Un centro de mando central utilizó tecnología audiovisual bidireccional para conectar a especialistas experimentados en cuidados intensivos con equipos clínicos de hospitales remotos. El servicio, siempre disponible, utilizaba aplicaciones de mensajería y llamadas telefónicas. Más tarde, la cobertura se amplió a otros pacientes médicos, neonatales, pediátricos y quirúrgicos que requerían cuidados críticos.

**Resultados:**

Entre junio de 2020 y diciembre de 2023, el servicio proporcionó 6014 teleconsultas para gestionar 1907 pacientes en 109 centros médicos, la mayoría en Pakistán y también en Afganistán, Kenia y República Unida de Tanzania. De los 1907 pacientes, 652 (34,4%) tenían COVID-19 y 1244 (65,6%) padecían otras enfermedades. La duración media de las teleconsultas fue de 14,5 min. De los 581 pacientes de los que se disponía de datos, 204 (35,1%) fallecieron. La regresión logística multinomial multivariante mostró que las probabilidades de muerte disminuían con el aumento del número de consultas (>3) por paciente (razón de probabilidades ajustada [aOR]: 0,28; intervalo de confianza del 95%, IC: 0,16-0,48), y con el aumento del número de recomendaciones (≥5) por consulta (aOR: 3,09; IC del 95%: 1,08-8,84).

**Conclusión:**

Nuestro servicio de teleasistencia en cuidados intensivos ayudó a tratar a pacientes en estado crítico en regiones donde antes no se disponía de cuidados intensivos. Aunque es necesario investigar el impacto clínico de este modelo, los responsables de tomar decisiones deberían considerar su uso para aumentar la provisión de cuidados críticos en entornos sanitarios remotos y con recursos limitados.

## ملخص

**الغرض:**

تطوير خدمة رعاية مركزة عن بعد، بحيث توفر الاستشارة عن بعد من نظير إلى نظير للأطباء في بيئات الرعاية الصحية النائية والمحدودة الموارد، لعلاج المرضى المصابين بأمراض خطيرة، وتقييم نتائج الخدمة.

**الطريقة:**

بدأت جامعة آغا خان وحدة العناية المركزة عن بعد لمرض فيروس كورونا 2019 (كوفيد 19) في عام 2020. استخدم مركز قيادة مركزي تقنية سمعية بصرية ثنائية الاتجاه، لربط أخصائيي الرعاية المركزة ذوي الخبرة بالفرق الإكلينيكية في بيئات المستشفيات النائية. تستخدم الخدمة، المتاحة دائمًا، تطبيقات المراسلة والمكالمات الهاتفية. تم توسيع التغطية لاحقًا لتشمل المرضى الآخرين من الأطباء، وحديثي الولادة، والأطفال، والجراحة، الذين يحتاجون إلى رعاية دقيقة.

**النتائج:**

قدمت الخدمة بين يونيو/حزيران 2020 وديسمبر/كانون ثاني 2023، عدد 6014 استشارة عن بعد، لإدارة حالات 1907 مريضًا، في 109 مرافق طبية، معظمها في باكستان وأفغانستان وكينيا وجمهورية تنزانيا المتحدة. ومن بين 1907 مريضًا، كان 652 (%34.4) مصابين بكوفيد 19، و1244 (%65.6) مصابين بأمراض أخرى. كان متوسط مدة الاستشارات عن بعد 14.5 دقيقة. ومن بين 581 مريضًا كانت بيانات نتائجهم متاحة، توفي 204 (%35.1). أظهر التحوّف اللوجستي متعدد الحدود ومتعدد المتغيرات أن احتمالات الوفاة انخفضت مع زيادة عدد الاستشارات (>3) لكل مريض (نسبة الاحتمالات المعدلة (aOR): 0.28؛ بفاصل ثقة مقداره %95: 0.16 إلى 0.48)، وزيادة عدد التوصيات (≥ 5) لكل استشارة (نسبة الاحتمالات المعدلة (aOR): 3.09؛ ‏بفاصل ثقة مقداره %95: 1.08 إلى 8.84).

**الاستنتاج:**

ساعدت خدمة الرعاية المركزة عن بعد لدينا في إدارة حالات المرضى المصابين بأمراض خطيرة في المناطق التي لم تكن الرعاية المركزة متاحة فيها من قبل. وفي حين أن هناك حاجة إلى إجراء بحث حول التأثير الإكلينيكي لهذا النموذج، إلا أنه يجب على صناع القرار النظر في استخدامه لزيادة توفير الرعاية الحرجة في البيئات الصحية النائية والمحدودة الموارد.

## 摘要

**目的:**

旨在为偏远地区和资源有限医疗护理机构的医生提供点对点重症监护远程看诊服务，以便其为重症患者开展治疗并评估服务效果。

**方法:**

阿迦汗大学于 2020 年成立了 2019 冠状病毒病 (COVID-19) 远程重症监护室。该中央指挥中心采用双向视听技术使偏远地区医院的临床团队能够与经验丰富的重症监护专家保持联系。该项服务可随时通过消息应用程序和电话通话提供。随后，服务覆盖范围延伸至其他需要重症监护的内科、新生儿、儿科和外科患者。

**结果:**

从 2020 年 6 月至 2023 年 12 月，该服务累计为 109 个医疗机构的 1,907 名患者提供 6,014 次远程看诊，这些医疗机构主要分布在阿富汗、巴基斯坦、肯尼亚和坦桑尼亚联合共和国。1907 名患者中，652 名患者 (34.4%) 曾感染 COVID-19，1,244 名患者 (65.6%) 患有其他疾病。远程看诊服务的平均时长为 14.5 分钟，在 581 例可获得疗效数据的患者中，204 例 (35.1%) 死亡。多因素多项逻辑回归分析显示死亡率随着每位患者看诊次数 (> 3) 的增加而下降，【调整后优势比 (aOR)：0.28；95% 置信区间 (CI)：0.16-0.48】，并且随着每次看诊的建议数 (≥ 5) 的增加而下降（aOR：3.09；95% CI：1.08-8.84 分）。

**结论:**

我们的远程重症监护服务帮助管理之前没有重症监护服务地区的重症患者。尽管我们需要对该模型的临床影响开展研究，但是决策者应考虑使用该模型，以提高偏远和资源有限地区医疗护理机构的重症监护服务覆盖率。

## Резюме

**Цель:**

Разработать службу телеинтенсивной терапии, предоставляющую одноранговые телеконсультации врачам в отдаленных и ограниченных в ресурсах медицинских учреждениях для лечения критически больных пациентов, и оценить результаты работы этой службы.

**Методы:**

В 2020 году в Университете Ага Хана открылось отделение телеинтенсивной терапии для больных коронавирусной инфекцией 2019 г. (COVID-19). Центральный командный пункт использовал двустороннюю аудиовизуальную технологию для подключения опытных специалистов по интенсивной терапии к клиническим бригадам в удаленных больницах. Этот сервис, который всегда был доступен, использовал в своей работе приложения для обмена сообщениями и телефонные звонки. Позже этот опыт был распространен на других медицинских, неонатальных, педиатрических и хирургических пациентов, нуждающихся в интенсивной терапии.

**Результаты:**

В период с июня 2020 года по декабрь 2023 года служба предоставила 6014 телеконсультаций для ведения 1907 пациентов в 109 медицинских учреждениях, в основном в Пакистане, а также в Афганистане, Кении и Объединенной Республике Танзания. Из 1907 пациентов у 652 (34,4%) диагностирован COVID-19, у 1244 (65,6%) – другие заболевания. Средняя продолжительность телеконсультаций составила 14,5 минуты. Из 581 пациента, по которым были получены данные о результатах, 204 (35,1%) скончались. По результатам многомерной мультиноминальной логистической регрессии вероятность смерти снижалась при увеличении количества консультаций (> 3) на пациента (скорректированное отношение шансов (aOR): 0,28; 95%-й ДИ: 0,16–0,48) и увеличении количества рекомендаций (≥ 5) на консультацию (aOR: 3,09; 95%-й ДИ: 1,08–8,84).

**Вывод:**

Благодаря службе телеинтенсивной терапии стало возможным лечение тяжелобольных пациентов в регионах, где ранее интенсивная терапия была недоступна. Несмотря на необходимость изучения клинического воздействия этой модели, ответственные лица должны рассмотреть возможность ее использования для повышения качества оказания интенсивной терапии в отдаленных и ограниченных в ресурсах медицинских учреждениях.

## Introduction

The effective functioning of intensive care units requires staffing with nurses, intensive care physicians and allied health professionals. Research suggests that adequate staffing is associated with reduced intensive care unit mortality and shorter length of stay.^1^^–^^5^ However, intensive care unit capacity, including beds, equipment and staff, can be limited and even non-existent in low- and middle-income countries. As of 2020, low-income countries had between 0.0 and 21.3 intensive care unit beds per 100 000 population, compared with 0.0 to 59.5 per 100 000 population in high-income countries.^3^ Globally, at least 96 countries and territories had fewer than 5.0 intensive care unit beds per 100 000 population.^3^ Most intensive care units in these places are in large urban referral hospitals, making it challenging for patients in rural areas to access critical care services.

In Pakistan, the reported critical care capacity is low for a population of about 227 million in 2020.^6^^,^^7^ A recent study found that only 36.8% (39/106) of hospitals in Pakistan employed accredited intensive care physicians.^8^ As in other low- and middle-income countries, most of Pakistan’s population (62.0%) lives in remote and rural locations and has limited access to appropriate medical care.^9^ This lack of access is because of the greater difficulty in providing care outside urban areas and the reluctance of physicians to work in these settings.^8^^–^^10^

Technological innovation has been used to meet the demand for intensive care unit services in low- and middle-income countries and overcome the shortage of intensive care specialists in many remote settings.^11^ Intensive care telemedicine (tele-intensive care) is a technology-based model designed to connect remotely located physicians with expert intensive care clinicians via technology such as video conferencing.^12^^–^^14^ Because tele-intensive care helps bedside physicians diagnose and treat the most acute hospital patients, this service has been associated with substantial reductions in mortality, shorter stays in hospital and the intensive care unit, and lower health-care costs.^12^^–^^14^ Traditional approaches to tele-intensive care are costly and require an advanced technology infrastructure which is usually proprietary, such as remotely accessible electronic medical records and live telemetry feeds for physiological data. Therefore, these systems are used more often in high-income countries^15^ than in low- and middle-income countries.^16^

Gaps in critical care capacity in low- and middle-income countries adversely affected the ability of health services to cope with surges in critically ill patients with coronavirus disease 2019 (COVID-19). Therefore, the Aga Khan University developed and implemented a tele-intensive care unit to address the lack of skilled personnel in resource-constrained health-care settings. The aim of this intervention was to bridge the gap between well-resourced and less-resourced intensive care facilities during the COVID-19 pandemic and beyond. The tele-intensive care unit uses two-way audiovisual communication through available devices, such as smartphones, coupled with computer systems to provide a collaborative, interprofessional care model focused on managing critically ill patients.^7^^,^^17^^–^^19^ In this study, we outline the processes involved in establishing tele-intensive care, and assess the effect of tele-intensive care on patient care and outcomes. 

## Methods

In June 2020, the Aga Khan University started the COVID-19 tele-intensive care unit project in collaboration with the federal governments of several countries and provincial governments in Pakistan. The objective of the tele-intensive care unit was to increase access of physicians in remote areas to intensive care experts who provided evidence-based guidance with the aim of improving patient outcomes by standardizing processes.^20^^–^^22^ This unit offered round-the-clock expert consultations for critically ill patients in Afghanistan, Kenya, Pakistan and United Republic of Tanzania. The Pakistani health ministry approved the project. The Aga Khan University Ethical Review Board approved the study (2020–4869–10701). 

### Development of the tele-intensive care unit

A project team consisting of a principal investigator, two co-principal investigators, a medical lead, a project manager, an assistant manager, and medical and research staff created a tele-intensive care central command centre to provide access to peer-to-peer consultation. 

#### Governance and management

The project team engaged with critical care stakeholders in public and private health-care sectors through the federal and provincial governments and health ministries across Pakistan to create the tele-intensive care hub. The federal and provincial governing bodies and the Aga Khan University signed memoranda of understanding outlining the scope, nature, limitations and expectations of the project, which enabled communication with public sector medical institutions across Pakistan. The government entities disseminated official notices about the intervention to applicable medical facilities to encourage their participation. Simultaneously, the project team ran a social media campaign announcing the tele-intensive care intervention targeting private sector institutions. All institutions willing to participate signed an agreement outlining roles, responsibilities, scope and liabilities. Each institution nominated a senior clinician as the focal person for coordination and stakeholder management. This person liaised with the tele-intensive care unit to identify local physicians working in intensive care who might ask for the consultations, facilitate implementation of consultations and overcome day-to-day operational challenges. Institutions outside Pakistan were approached about participating in the second year of the project. 

#### Staffing and training

The Aga Khan University assembled a multidisciplinary team of up to 16 tele-intensive care consultants. The team included anaesthesiologists, intensive care physicians, internal medicine physicians, neonatologists and paediatricians. The project team developed monthly workflow schedules and rotations, and consultants were paid a small stipend for each consultation. Additionally, the project leads recruited recent medical graduates who were trained as tele-intensive care officers to facilitate communication between the remote physicians and the tele-intensive care consultants. Details of their training are published elsewhere.^23^ These officers continuously monitored the telephones and WhatsApp (Meta, Menlo Park, United States of America) groups for consultation requests, documented the patient care recommended from the consultations and maintained the tele-intensive care patient registry. A manager oversaw stakeholder management, external and internal communication, data management and the operational processes.

#### Tele-intensive care unit framework

The tele-intensive care officers were located either inside or outside the command centre. They connected the tele-intensive care consultants to medical facilities requesting consultations, or arranged remote consultation through computers or mobile devices with videoconferencing capability from any location with internet access.

Consultative services could be provided at predetermined times at the convenience of the requesting physicians and tele-intensive care consultant (scheduled-care model). Alternatively, teleconsultations were prompted by an alert through which the requesting physician shared the patient’s condition with the tele-intensive care officer to get an immediate response (responsive-care model). A hotline number was assigned for such calls.

#### Teleconsultation process

**Fig. 1:**
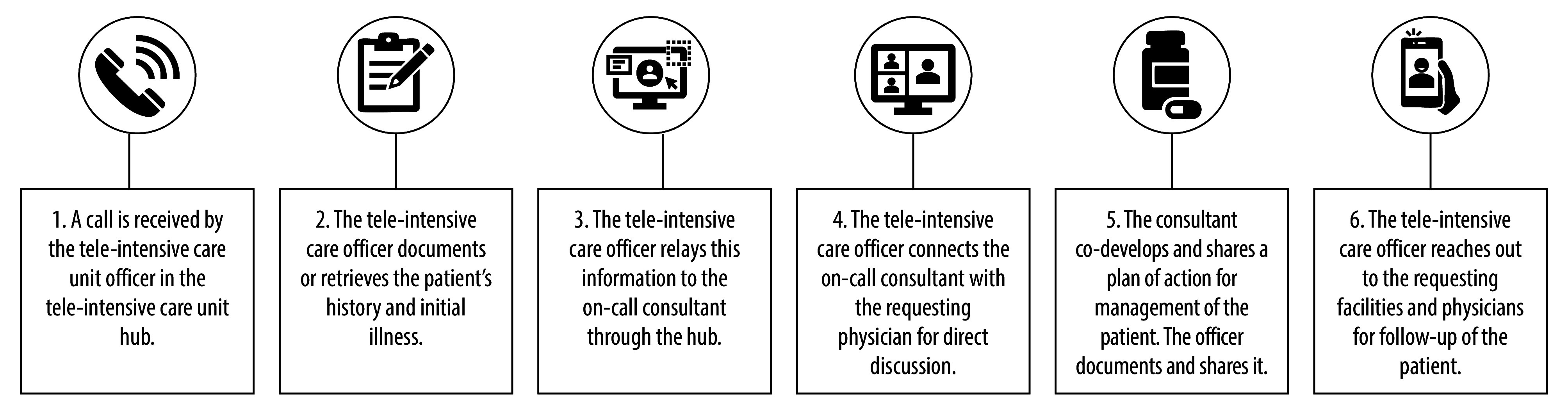
Steps in the tele-intensive care unit consultation process

Remote physicians initiated teleconsultation requests through text messages or telephone calls (Fig. 1). Thereafter, WhatsApp, telephones, mobile telephones and Zoom (Zoom Communications Inc., San Jose, USA) platforms were used for communication as they were readily available to the remote physicians. Relevant patient medical data, such as radiographs, laboratory tests, medications, ventilation management and acute adverse events in the past 24 hours, were sent from the remote physician to the tele-intensive care officer. The officer then arranged a three-way teleconsultation between themself, the requesting physician and the tele-intensive care consultant.

#### Patients

The patients referred for tele-intensive care were inpatients in the high-dependency or intensive care unit of the remote facilities whom the bedside physician thought needed critical care consultation. Initially, consultations were provided for COVID-19 patients; however, after 12 months, coverage was broadened to include other surgical, medical, neonatal and paediatric critical care patients.

#### Promotion and dissemination

Social media platforms, the Aga Khan University’s official webpage and e-flyers distributed via emails (to people who had previously registered for any education course offered by the university) were used to raise awareness of the tele-intensive care service and hotline. We also initially used site visits to medical facilities across Pakistan to promote the service and build relationships.

#### Data management

The tele-intensive care officer provided a summary of the consultation, including patient assessment and a management plan recommended by tele-intensive care consultants to the requesting remote physician. Details of the teleconsultation (i.e. patient demographics, admission symptoms, investigations and medications discussed, management recommendations, and consultation times and method) were documented in the tele-intensive care patient registry. Investigations discussed were categorized into haematology, biochemistry, radiology and COVID-19-related tests. Management recommendations were categorized into diagnostic interventions (e.g. recommendations for further testing) and therapeutic interventions (e.g. pharmaceutical and critical care supportive management). Only tele-intensive care officers and their supervisors had permission to access and edit these data. Patients were assigned a unique medical record number and data were de-identified. The tele-intensive care consultants addressed any subsequent queries through text-based communications, which were recorded by tele-intensive care officers. The officers conducted follow-ups after 24–48 hours, with further follow-up teleconsultations scheduled if required.

#### Funding

The project was funded by in-kind funding by the Aga Khan University and independent grants from agencies listed in the funding section. The university support included space for the hub and faculty time. The agency funding covered all programmatic costs including equipment, project personnel, required travel and meetings and consultant stipends, and educational content development and delivery. The consultation service was free for the remote institutions.

### Data analysis

We used an observational quantitative design to evaluate the care provision processes and assess outcomes of patients. The project team retrieved data for the number of new COVID-19 patients in Pakistan from the Government of Pakistan’s official website. We present data as numbers and percentages for categorical variables. We did a stratification analysis of intensive care unit outcomes. We used multivariate multinomial logistic regression analysis to assess factors associated with intensive care unit outcome. *P* < 0.05 was considered statistically significant. The reference variable was discharged home. We used Excel, version 2005 (Microsoft, Redmond, USA) and R 4.1.2 (R Foundation, Vienna, Austria) for data analysis. For the costing analysis, we used actual incurred costs for programmatic start-up, and fixed and variable operational costs based on United States dollars (US$) of April 2023.

## Results

**Fig. 2:**
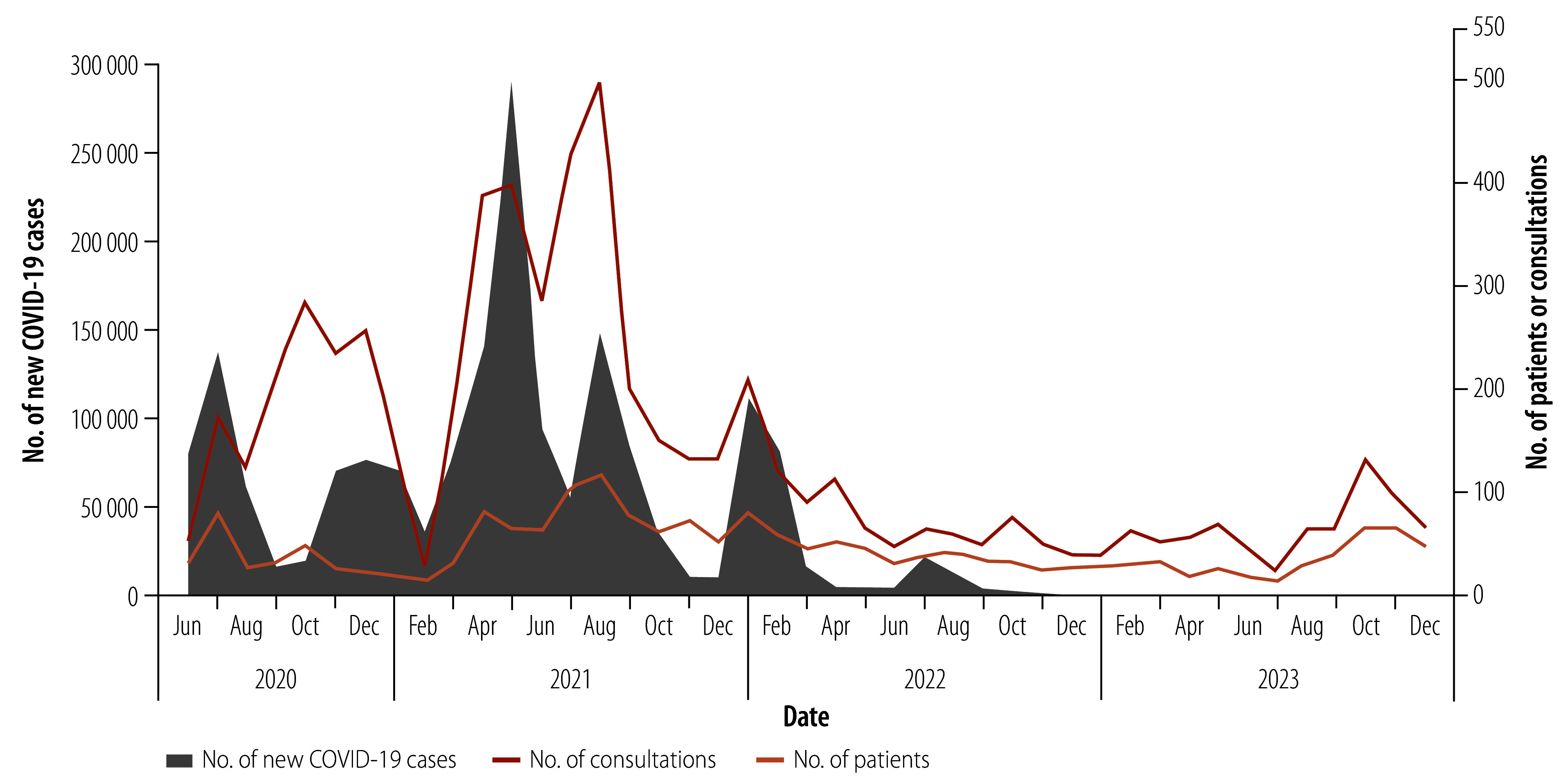
Number of patients and consultations and new COVID-19 cases reported in Pakistan, by month and year, June 2020 to December 2023

The tele-intensive care unit provided 6014 consultations for 1907 patients from 1 June 2020 to 31 December 2023, with an average of 139.9 consultations per month. A total of 1138 (59.7%) patients received one consultation and 769 (40.3%) received more than one. Most patients referred for tele-intensive care were male (1016: 53.6%) and lived in Pakistan (1858: 98.3%). Of the 5291 consultations conducted with known type of consultation, 2728 (51.9%) were scheduled and 2533 (48.1%) were responsive. Of the 652 (34.4%) patients presenting with COVID-19, 344 had severe symptoms and 172 had critical symptoms;^24^^,^^25^ 1244 (65.6%) did not have COVID-19 (Table 1). The highest number of teleconsultations was in August 2021 (Fig. 2; online repository).^26^ The most common active medical issue for which consultations were requested were respiratory conditions (5515/8436 requests; 65.4%) and neurological disorders (634/8436 requests; 7.5%; Table 2).

**Table 1:** Characteristics of consultations and patients referred to the tele-intensive care unit, June 2020 to December 2023

Variable	No. (%)^a^
**Total no. of consultations**	6014
**Type of consultation**	
Scheduled	2728 (51.9)
Responsive	2533 (48.1)
Unknown	753 (12.5)
**Total no. of patients**	1907
**No. of consultations per patient**	
1	1138 (59.7)
> 1	769 (40.3)
**Sex of patient**	
Male	1016 (53.6)
Female	878 (46.4)
Unknown	13 (0.7)
**Age, in years**	
< 18	397 (21.6)
18–38	476 (25.9)
39–58	395 (21.5)
59–78	458 (24.9)
79–98	112 (6.1)
> 98	2 (0.1)
Unknown	67 (3.5)
**Location of health facility**	
Afghanistan	9 (0.5)
Kenya	8 (0.4)
Pakistan	1858 (98.3)
Baluchistan	5 (0.3)
Gilgit Baltistan	353 (19.0)
Khyber Pakhtunkhwa	351 (18.9)
Chitral	88 (4.7)
Punjab	713 (38.4)
Sindh	348 (18.7)
United Republic of Tanzania	16 (0.8)
Unknown	16 (0.8)
**Diagnosis on admission**	
COVID-19	652 (34.4)
Asymptomatic	37 (5.7)
Non-severe	76 (11.7)
Severe	344 (52.8)
Critical	172 (26.4)
Suspected	23 (3.5)
Other	1244 (65.6)
Unknown	11 (0.6)

^a^ Percentages are calculated from the total sample minus the number of unknowns. The unknown percentages are calculated from the total sample.

COVID-19: coronavirus disease 2019.

**Table 2:** Types of medical problem for which remote physicians requested tele-intensive care consultations, Afghanistan, Kenya, Pakistan, United Republic of Tanzania, June 2020 to December 2023

Type of medical problem^a^	No. (%) (*n* = 8436)
Cardiological	454 (5.4)
Dermatological	96 (1.14)
Endocrine	176 (2.1)
Ear, nose and throat	15 (0.2)
Gastrointestinal	286 (3.4)
Gynaecological	6 (0.1)
Haematological	144 (1.7)
Infectious diseases	387 (4.6)
Metabolic disorders	291 (3.4)
Musculoskeletal	47 (0.6)
Renal	259 (3.1)
Neurological	634 (7.5)
Psychological	59 (0.7)
Respiratory	5515 (65.4)
Other	67 (0.8)

^a^ A patient could have had more than one medical problem.

Teleconsultations were provided to 109 medical institutions, 105 in Pakistan, three in East Africa (two in Kenya, one in United Republic of Tanzania) and one in Afghanistan. The average number of consultations per patient was 3.2 in Pakistan, 2.4 in East Africa and 2.1 in Afghanistan. The institutions included three primary care facilities, 47 secondary care facilities, 51 tertiary care facilities and one temporary COVID-19 testing and vaccination facility; for seven facilities the type was unknown. Of the 109 facilities, 29 were in rural areas, 77 were in urban areas, with the location of three facilities unknown.^27^

The total consultation call duration was 77 981 minutes, mean duration of 14.53 minutes/call (online repository).^26^ For the 6014 consultations, voice calls were the most frequently used consultation method (4368; 72.6%; online repository).^26^

The tele-intensive care consultants reviewed 16 166 investigations (laboratory tests and radiological images), with a mean of 8.5 investigations reviewed per patient and 2.7 investigations per consultation. The investigations were related to biochemistry (9310; 57.6%), haematology (3499; 21.6%), radiology (2233; 13.8%), COVID-19 investigations (591; 3.7%), microbiology (484; 3.0%) and other investigations (49; 0.3%). The most frequent specific investigations were complete blood count (2734), creatinine level (2013) and arterial blood gases (1971; online repository).^26^

Tele-intensive care consultants made 37 904 management recommendations, with a mean of 19.9 recommendations per patient and 6.3 recommendations per consultation. The most management recommendations (3897) were provided during August 2021. The most frequently recommended management advice related to therapeutic techniques to manage infectious diseases and infection control (6752 recommendations; online repository).^26^

Outcome data were available for 581 (30.5%) patients, 204 (35.1%) of whom died (online repository).^26^ Multivariate multinomial logistic regression analysis showed no significant relationship between patient outcome and sex. The odds of death decreased significantly with increased number of consultations (> 3 consultations) per patient (adjusted odds ratio, aOR: 0.28; 95% confidence interval, CI: 0.16–0.48). Patients who received more than five recommendations had significantly greater odds of death than patients with fewer recommendations (aOR: 3.09; 95% CI: 1.08–8.84; Table 3).

**Table 3:** Factors associated with intensive care unit outcome

Variable	aOR (95% CI)			
	Discharged from intensive care unit	Died	Left health-care facility against medical advice	Transferred
**Sex**				
Female	1.00 (reference)	1.00 (reference)	1.00 (reference)	1.00 (reference)
Male	0.98 (0.58–1.70)	1.26 (0.80–1.98)	0.77 (0.27–2.18)	1.04 (0.57–1.92)
**No. of consultations**				
1	1.00 (reference)	1.00 (reference)	1.00 (reference)	1.00 (reference)
2–3	0.57 (0.27–1.20)	0.76 (0.40–1.48)	0.88 (0.19–4.20)	0.46 (0.20–1.10)
> 3	0.20 (0.10–0.37)	0.28 (0.16–0.48)	0.43 (0.12–1.57)	0.23 (0.11–0.46)
**No. of laboratory tests**				
0	1.00 (reference)	1.00 (reference)	1.00 (reference)	1.00 (reference)
1	1.16 (0.32–4.31)	0.91 (0.36–2.32)	0.28 (0.03–3.12)	0.66 (0.18–2.37)
2–4	2.02 (0.65–6.25)	1.29 (0.58–2.84)	0.61 (0.13–2.88)	1.13 (0.40–3.22)
≥ 5	2.97 (0.94–9.27)	1.38 (0.62–3.12)	0.44 (0.09–2.24)	0.95 (0.33–2.81)
**Mean time of consultation (minutes)**				
< 15	1.00 (reference)	1.00 (reference)	1.00 (reference)	1.00 (reference)
15–30	0.96 (0.51–1.78)	1.15 (0.69–1.91)	1.30 (0.41–4.07)	0.89 (0.43–1.83)
> 30	0.15 (0.02–1.34)	0.85 (0.32–2.30)	0.70 (0.07–7.08)	0.66 (0.15–2.84)
**No. of recommendations**				
0	1.00 (reference)	1.00 (reference)	1.00 (reference)	1.00 (reference)
1	3.23 (0.51–20.00)	1.71 (0.38–7.59)	4.35 × 10^−9^ (8.25 × 10^−10^–8.30 × 10^−9^)	1.55 (0.18–13.15)
2–4	1.63 (0.38–6.99)	2.50 (0.86–7.32)	0.22 (0.02–2.96)	1.63 (0.38–7.18)
≥ 5	1.43 (0.34–6.01)	3.09 (1.08–8.84)	1.49 (0.23–9.49)	1.65 (0.38–7.10)
**Diagnosis on admission**				
Asymptomatic COVID-19	1.00 (reference)	1.00 (reference)	1.00 (reference)	1.00 (reference)
Critical COVID-19	0.27 (0.01–4.75)	2.55 (0.30–21.50)	48 057.44 (17 950.98–128 656.90)	0.36 (0.02–5.76)
Severe COVID-19	0.97 (0.07–12.79)	0.94 (0.12–7.62)	8 951.41 (3 547.05–22 589.99)	0.55 (0.04–7.02)
Non-severe COVID-19	0.20 (0.01–5.70)	0.13 (0.01–2.47)	1.0 × 10^−5^ (1.4 × 10^−6^–6.28 × 10^−5^)	0.19 (0.01–5.23)
Non-COVID-19 illness	2.28 (0.19–30.73)	1.96 (0.24–16.11)	23 515.98 (9 120.56–60 632.37)	1.58 (0.12–20.24)

CI: confidence interval; COVID-19: coronavirus disease 2019; aOR: adjusted odds ratio.

Note: We used a multivariate multinomial logistic regression to calculate aOR. Discharged home was the reference variable.

Initial capital costs of the project, including computers, mobile telephones, accessories such as webcams and headsets, and a Zoom account were US$ 9467. Ongoing fixed operational costs, including personnel salaries (physician project lead, management and administrative staff, and tele-intensive care officers) and consumables, were aggregated for a typical month and came to US$ 168.88 per day. Ongoing variable operational costs, mainly for consultant stipends, were US$ 18.20 per patient.

## Discussion

Telemedicine can facilitate patient stabilization by providing immediate virtual assistance while using the knowledge and skills of the bedside physicians.^12^^,^^20^ Peer-to-peer telemedicine has shown success, particularly in high-income countries, with reduced intensive care and hospital stay and mortality reported.^20^^,^^28^^,^^29^ Research also shows that a lack of critical care staff to meet needs can be bridged by providing teleconsultation services.^30^^–^^32^ However, previous efforts have required extensive and often costly technological resources to deliver these services.^7^^,^^33^^–^^35^ Our results show that a tele-intensive care unit was able to reach remote intensive care facilities using existing telecommunication and information technology. They also demonstrate the feasibility of using this model in low- and middle-income countries to provide consultative services to facilities with limited resources, trained critical care staff and infrastructure.

During the COVID-19 pandemic, the number of teleconsultations mirrored the national COVID-19 numbers and waves. After the pandemic, there was an initial lull in teleconsultations but these began to rise again in mid-2023. These consultations were likely for other kinds of critically ill patients and as a result of wider recognition of this service. Bedside physicians most frequently requested teleconsultations for management strategies for respiratory complications. Severe and critical diseases can lead to respiratory failure and acute respiratory distress syndrome, which require rapid implementation of interventions such as intubation and mechanical ventilation that many physicians are not comfortable using.^36^ This observation aligns with the fact that 79.1% (516/652) of the patients with COVID-19 presented with severe or critical symptoms which generally require advanced respiratory support. Severe respiratory problems remained the leading reason for consultation after the pandemic ended.

Tele-intensive care has reduced intensive care and hospital mortality, further endorsing the validity of these services.^16^^,^^20^^–^^22^^,^^28^^,^^29^ In the United States, tele-intensive care services reduced the hospital mortality from 13.6% (208/1529) before the intervention to 11.8% (562/4761) after implementation.^1^ Our service recorded a 35.1% (204/581) mortality (among patients for whom outcome data were available); and our multivariate multinomial logistic regression analyses indicated that the more teleconsultations provided per patient, the greater the likelihood of patient survival. This mortality was similar to that in critically ill patients in a tertiary care facility in metropolitan Pakistan in a 90-day study (39.4%; 67/170).^37^ This finding is noteworthy given the differences between a teleconsultation model and in-person model for patient care, as well as the differences in resources between the two models. A teleconsultation model can also be more easily scaled up than one based on in-person care. While further study about causality and clinical impact of this model is needed, decision-makers should consider its use to increase provision of critical care in resource-constrained settings.

Our service offered peer-to-peer patient management supported 109 facilities in Afghanistan, Kenya, Pakistan and the United Republic of Tanzania. A few primary care facilities, which usually do not have intensive care units, used our services when COVID-19 started, as they were the first health-system access point for ill patients. To broaden the reach and impact of such a teleconsultation service, primary care facilities should be approached for collaboration as they often provide initial acute care and referrals for critically ill patients. Measures might be needed to provide the necessary technology, as many do not have access to high-speed internet and may not have the technology for virtual consultations.

A limitation of our study was the potential for selection bias towards minimizing the impact on patient outcomes, given that bedside physicians might have used this tele-intensive care service for their sickest patients, potentially leading to a higher mortality rate. Additionally, the main objective of the service was provision of tele-consultations for critically ill patients to support staff during the pandemic and beyond; as such, patient outcomes were not the focus of the project. Therefore, not all patients could be followed up, and less than one third of the patients for whom consultations were provided were included in our analysis. Since most of the patients lost to follow-up had only one consultation, the effect on the outcomes of critically ill patients would be limited.

The tele-intensive care service faced various implementation and sustainability challenges. We conducted a preliminary study (unpublished data) of bedside physicians’ perception of tele-intensive care. Despite offering free peer-to-peer consultations and receiving positive feedback during COVID-19 surges, some physicians, particularly senior doctors, perceived them as a threat to their practices and autonomy, and did not allow junior doctors in their units to start consultations. Furthermore, these physicians were reluctant to disseminate information about the service in their units. Additionally, most patients had only one consultation. Another challenge was obtaining patient outcomes after the patient became stable or was discharged from the intensive care unit. Because tele-intensive care services were provided free-of-charge for patients and bedside physicians received no financial support for teleconsultation, these physicians might have seen daily consultation as an additional burden. Consequently, they may have been reluctant to provide extensive documentation to the Aga Khan University and shared only limited information.

To address these challenges, we held meetings with hospital staff and maintained constant engagement through educational activities and e-mentoring for bedside physicians. These efforts resulted in sustained engagement with several institutes. Indeed, 31 of the 109 institutes are still using our tele-intensive care services. Our medical officers also conduct daily follow-up calls after teleconsultations to assess patient outcomes and promote further consultations for each patient. Additionally, they document all patient health information during audioconferencing to reduce the documentation burden on the bedside physicians.

The COVID-19 pandemic increased the use of telemedicine to deliver care to remote and resource-constrained facilities. Our tele-intensive care service has successfully helped manage critically ill patients since June 2020 in regions where tele-intensive care had not previously been used. This project has continued due to its low implementation cost per patient, continuous availability of intensive care doctors, and use of existing telecommunication and information technology infrastructure. Future research and coordination are necessary to increase access to the internet and technology required for teleconsultations and to determine the effect of the project on patient mortality. 

## References

[1] Lilly CM, Cody S, Zhao H, Landry K, Baker SP, McIlwaine J, et al. University of Massachusetts Memorial Critical Care Operations Group. Hospital mortality, length of stay, and preventable complications among critically ill patients before and after tele-ICU reengineering of critical care processes. JAMA. 2011 Jun 1;305(21):2175–83. 10.1001/jama.2011.697 21576622

[2] Ervin JN, Kahn JM, Cohen TR, Weingart LR. Teamwork in the intensive care unit. Am Psychol. 2018 73(4);468–77. 10.1037/amp0000247 29792461 PMC6662208

[3] Ma X, Vervoort D. Critical care capacity during the COVID-19 pandemic: global availability of intensive care beds. J Crit Care. 2020 Aug;58:96–7. 10.1016/j.jcrc.2020.04.012 32408107 PMC7194590

[4] Kim MM, Barnato AE, Angus DC, Fleisher LA, Kahn JM. The effect of multidisciplinary care teams on intensive care unit mortality. Arch Intern Med. 2010 Feb 22;170(4):369–76. 10.1001/archinternmed.2009.521 20177041 PMC4151479

[5] Pronovost PJ, Angus DC, Dorman T, Robinson KA, Dremsizov TT, Young TL. Physician staffing patterns and clinical outcomes in critically ill patients: a systematic review. JAMA. 2002 Nov 6;288(17):2151–62. 10.1001/jama.288.17.2151 12413375

[6] Hashmi M, Taqi A, Memon MI, Ali SM, Khaskheli S, Sheharyar M, et al. A national survey of critical care services in hospitals accredited for training in a lower-middle income country: Pakistan. J Crit Care. 2020 Dec;60:273–8. 10.1016/j.jcrc.2020.08.017 32942162 PMC7441021

[7] Population, total – Pakistan. Washington, DC: World Bank; 2020. Available from: https://data.worldbank.org/indicator/SP.POP.TOTL?locations=PKe [cited 2024 Nov 2].

[8] Khan MA, Shahbaz H, Noorali AA, Ehsan AN, Zaki M, Asghar F, et al. Disparities in adult critical care resources across Pakistan: findings from a national survey and assessment using a novel scoring system. Crit Care. 2022 Jul 11;26(1):209. 10.1186/s13054-022-04046-5 35818054 PMC9272593

[9] Rural population (% of total population). Washington, DC: World Bank; 2023. Available from: https://data.worldbank.org/indicator/SP.RUR.TOTL.ZS [cited 2024 Jun 18].

[10] Farooq U, Ghaffar A, Narru IA, Khan D, Irshad R. Doctors' perception about staying in or leaving rural health facilities in District Abbottabad. J Ayub Med Coll Abbottabad. 2004 Apr-Jun;16(2):64–9. 15455622

[11] Rosenfeld BA, Dorman T, Breslow MJ, Pronovost P, Jenckes M, Zhang N, et al. Intensive care unit telemedicine: alternate paradigm for providing continuous intensivist care. Crit Care Med. 2000 Dec;28(12):3925–31. 10.1097/00003246-200012000-00034 11153637

[12] Becker C, Frishman WH, Scurlock C. Telemedicine and tele-ICU: the evolution and differentiation of a new medical field. Am J Med. 2016 Dec;129(12):e333–4. 10.1016/j.amjmed.2016.05.045 27576079

[13] Watanabe T, Ohsugi K, Suminaga Y, Somei M, Kikuyama K, Mori M, et al. An evaluation of the impact of the implementation of the tele-ICU: a retrospective observational study. J Intensive Care. 2023 Mar 7;11(1):9. 10.1186/s40560-023-00657-4 36882878 PMC9989570

[14] Grundy BL, Jones PK, Lovitt A. Telemedicine in critical care: problems in design, implementation, and assessment. Crit Care Med. 1982 Jul;10(7):471–5. 10.1097/00003246-198207000-00014 7083874

[15] Koenig MA. Telemedicine in the ICU. Cham: Springer; 2019. 10.1007/978-3-030-11569-2

[16] Williams D Jr, Lawrence J, Hong YR, Winn A. Tele–ICUs for COVID–19: a look at national prevalence and characteristics of hospitals providing teleintensive care. J Rural Health. 2021 Jan;37(1):133–41. 10.1111/jrh.12524 33030761 PMC7675587

[17] Reynolds HN, Bander J, McCarthy M. Different systems and formats for tele-ICU coverage: designing a tele-ICU system to optimize functionality and investment. Crit Care Nurs Q. 2012 Oct-Dec;35(4):364–77. 10.1097/CNQ.0b013e318266bc26 22948371

[18] Reynolds HN, Rogove H, Bander J, McCambridge M, Cowboy E, Niemeier M. A working lexicon for the tele-intensive care unit: we need to define tele-intensive care unit to grow and understand it. Telemed J E Health. 2011 Dec;17(10):773–83. 10.1089/tmj.2011.0045 22029748

[19] Davis TM, Barden C, Dean S, Gavish A, Goliash I, Goran S, et al. American telemedicine association guidelines for teleICU operations. Telemed J E Health. 2016 Dec;22(12):971–80. 10.1089/tmj.2016.0065 27508454

[20] Hawkins HA, Lilly CM, Kaster DA, Groves RH Jr, Khurana H. ICU telemedicine comanagement methods and length of stay. Chest. 2016 Aug;150(2):314–9. 10.1016/j.chest.2016.03.030 27048869

[21] Mackintosh N, Terblanche M, Maharaj R, Xyrichis A, Franklin K, Keddie J, et al. Telemedicine with clinical decision support for critical care: a systematic review. Syst Rev. 2016 Oct 18;5(1):176. 10.1186/s13643-016-0357-7 27756376 PMC5070369

[22] Khunlertkit A, Carayon P. Contributions of tele-intensive care unit (Tele-ICU) technology to quality of care and patient safety. J Crit Care. 2013 Jun;28(3):315.e1–12. 10.1016/j.jcrc.2012.10.005 23159139

[23] Latif A, Zaki M, Shahbaz H, Hussain SA, Daudpota AA, Imtiaz B, et al. Mass online training of health care workers during COVID-19: approach, impact, and outcomes for over 10 000 health care providers. Public Health. 2024 Aug;233:193–200. 10.1016/j.puhe.2024.05.006 38941682 PMC11283886

[24] Bhimraj A, Morgan RL, Shumaker AH, Baden LR, Cheng VC, Edwards KM, et al. Infectious Diseases Society of America Guidelines on the Treatment and Management of Patients With COVID-19 (September 2022). Clin Infect Dis. 2024 Jun 27;78(7):e250–349. 10.1093/cid/ciac724 36063397 PMC9494372

[25] Cascella M, Rajnik M, Aleem A, Dulebohn SC, Di Napoli R. Features, evaluation, and treatment of coronavirus (COVID-19). Treasure Island: StatPearls Publishing; 2024. 32150360

[26] Latif A, Atiq H, Zaki M, Hussain SA, Ghayas A, Shafiq O, et al. A pragmatic approach for implementation of peer-to-peer tele-consultative services in low-resource environments to deliver critical care at scale. Supplementary material [online repository]. Frankfurt: Open Science Framework; 2024. 10.17605/OSF.IO/6XAY9

[27] Population census 2023 [internet]. Islamabad: Pakistan Bureau of Statistics; 2023. Available from: https://www.pbs.gov.pk/content/final-results-census-2017-0 [cited 2024 Oct 10].

[28] Lilly CM, Thomas EJ. Tele-ICU: experience to date. J Intensive Care Med. 2010 Jan-Feb;25(1):16–22. 10.1177/0885066609349216 19752038

[29] Macedo BR, Garcia MVF, Garcia ML, Volpe M, Sousa MLA, Amaral TF, et al. Implementation of tele-ICU during the COVID-19 pandemic. J Bras Pneumol. 2021 Apr 30;47(2):e20200545. 10.36416/1806-3756/e20200545 33950091 PMC8332846

[30] Raheja R, Unnikrishnan DC, Anand S, Joshi D, Raman D. Feasibility of tele-ICU augmented cardiopulmonary resuscitation in a resource limited setting: a pilot study. Resuscitation. 2019 May;138:208–9. 10.1016/j.resuscitation.2019.02.044 30872070

[31] Babatunde A, Abdulazeez A, Adeyemo E, Uche-Orji C, Saliyu A. Telemedicine in low- and middle-income countries: closing or widening the health inequalities gap. Eur J Environ Public Health. 2021;5(2):em0075. 10.21601/ejeph/10777

[32] Atta-ur-Rahman, Salam MH, Jamil S, Virtual clinic: a telemedicine proposal for remote areas of Pakistan. In: Proceedings of the 2013 Third World Congress on Information and Communication Technologies (WICT 2013); 15–18 Dec 2013; Hanoi, Viet Nam. Piscataway: Institute of Electrical and Electronics Engineers; 2013.

[33] Dowler S, Crosbie K, Thompson S, Drucker E, Jackson C. Telemedicine utilization trends during the COVID-19 public health emergency. N C Med J. 2021 Jul-Aug;82(4):255–8. 10.18043/ncm.82.4.255 34230176

[34] James N, Menzies M. Trends in COVID-19 prevalence and mortality: a year in review. Physica D. 2021 Nov;425:132968. 10.1016/j.physd.2021.132968 34121785 PMC8183049

[35] Pakistan humanitarian situation report No. 28. New York: United Nations Children’s Fund; 2021. Available from: https://www.unicef.org/documents/pakistan-humanitarian-situation-report-31-august-2021 [cited 2024 Nov 2].

[36] Tobin MJ. Basing respiratory management of COVID-19 on physiological principles. New York: American Thoracic Society; 2020. pp. 1319–20. 10.1164/rccm.202004-1076ED PMC7258630 32281885

[37] Sarfaraz S, Shaikh Q, Saleem SG, Rahim A, Herekar FF, Junejo S, et al. Determinants of in-hospital mortality in COVID-19; a prospective cohort study from Pakistan. PLoS One. 2021 May 27;16(5):e0251754. 10.1371/journal.pone.0251754 34043674 PMC8158897

